# Serum AGE/RAGEs as potential biomarker in idiopathic pulmonary fibrosis

**DOI:** 10.1186/s12931-018-0924-7

**Published:** 2018-11-08

**Authors:** Carlos Machahua, Ana Montes-Worboys, Lurdes Planas-Cerezales, Raquel Buendia-Flores, Maria Molina-Molina, Vanesa Vicens-Zygmunt

**Affiliations:** 1 Pneumology Research Group, IDIBELL, L’Hospitalet de Llobregat, Barcelona, Spain; 2 Biomedical Research Network Centers in Respiratory Diseases (CIBERES), Barcelona, Spain; 3 Unit of Interstitial Lung Diseases, Department of Pneumology, University Hospital of Bellvitge, C. Feixa Llarga sn., 08907 L’Hospitalet de Llobregat, Barcelona, Spain

**Keywords:** AGEs, RAGEs, IPF, Biomarker

## Abstract

**Background:**

The soluble receptor for advanced glycation end-products (sRAGE) has been suggested that it acts as a decoy for capturing advanced glycation end-products (AGEs) and inhibits the activation of the oxidative stress and apoptotic pathways. Lung AGEs/sRAGE is increased in idiopathic pulmonary fibrosis (IPF). The objective of the study was to evaluate the AGEs and sRAGE levels in serum as a potential biomarker in IPF.

**Methods:**

Serum samples were collected from adult patients: 62 IPF, 22 chronic hypersensitivity pneumonitis (cHP), 20 fibrotic non-specific interstitial pneumonia (fNSIP); and 12 healthy controls. In addition, 23 IPF patients were re-evaluated after 3-year follow-up period. Epidemiological and clinical features were recorded: age, sex, smoking habits, and lung function. AGEs and sRAGE were evaluated by ELISA, and the results were correlated with pulmonary functional test values.

**Results:**

IPF and cHP groups presented a significant increase of AGE/sRAGE serum concentration compared with fNSIP patients. Moreover, an inverse correlation between AGEs and sRAGE levels were found in IPF, and serum sRAGE at diagnosis correlated with FVC and DLCO values. Additionally, changes in serum AGEs and sRAGE correlated with % change of FVC, DLCO and TLC during the follow-up. sRAGE levels below 428.25 pg/ml evolved poor survival rates.

**Conclusions:**

These findings demonstrate that the increase of AGE/sRAGE ratio is higher in IPF, although the levels were close to cHP. AGE/sRAGE increase correlates with respiratory functional progression. Furthermore, the concentration of sRAGE in blood stream at diagnosis and follow-up could be considered as a potential prognostic biomarker.

**Electronic supplementary material:**

The online version of this article (10.1186/s12931-018-0924-7) contains supplementary material, which is available to authorized users.

## Background

Idiopathic pulmonary fibrosis (IPF) is the most frequent form of fibrotic interstitial lung diseases (ILDs), which presents a variable outcome and is generally fatal within 2–4 years from diagnosis [1]. Histologically, IPF is characterized by an usual interstitial pneumonia (UIP) pattern; where honeycombing areas with collagen deposition and fibroblast foci are situated next to structurally preserved areas [2]. Advances in the knowledge of the pathogenesis have been focused in integrating the different factors involved in IPF with the purpose of improving the diagnosis, management and treatment [3].

In clinical practice, some fibrosing lung entities such as chronic hypersensitivity pneumonitis (cHP) and fibrotic nonspecific interstitial pneumonia (fNSIP) represent a challenge in the differential diagnosis for IPF as there are clinical and radiological similarities [4–8]. Even after an invasive procedure such as lung biopsy, it occasionally remains difficult to differentiate IPF from cHP [9, 10]. Though some features may be similar to IPF [11], the prognosis is better for fNSIP and cHP, and the treatment differs [12]. The proteomic analysis could be a useful tool to differentiate between these entities and to reach an accurate diagnosis, attempting to avoid invasive diagnostic procedures [13]. In the last decades, serum and broncoalveolar biological markers have been extensively studied in IPF for diagnosis and prognosis [14–17]. However, despite the progress in identifying new biomolecules involved in IPF pathogenesis, none improve the differential diagnosis and monitoring of the clinical course [18].

Recent studies have reported the implication of advanced glycation end-products (AGEs) and their receptor (RAGE) with IPF and other fibrotic ILDs [19]. In fact, a previous study within our group reported an AGE-RAGE imbalance in lung tissue from IPF patients compared to controls [20]. The RAGE is an immunoglobulin superfamily protein [21] implicated in the maintenance of alveolar structures in lung tissue [22] and the development and differentiation of Type I pneumocytes [23]. Its soluble form (sRAGE) is secreted directly to the extracellular matrix (ECM) through loss of its transmembrane region by cleavage [24, 25]. This is of particular interest because sRAGE has been proposed as an AGE decoy [26]. Some groups have suggested that sRAGE bind AGEs, blocking the cell signaling pathway of AGEs or attenuating their effects [27–29]. AGEs are the result of non-enzymatic reactions between a reducing sugar monosaccharides with a free amino group of proteins [30], widely studied in oxidative stress, inflammation and aging [31, 32]. AGEs also contribute to abnormal wound healing by acting on signaling pathway in cells [33] and ECM protein cross-links [34]; these changes may alter the physicochemical properties of collagen fibers modifying the tissue stiffness [35]. Given that RAGE is highly expressed in lung tissue [36], sRAGE could be a possible serum or plasma biomarker to study the pulmonary disorder.

Some studies of blood [37] and bronchoalveolar lavage [38] indicate a correlation between lung injury and the levels of sRAGE [39–41]. Nevertheless, there is scarce information about the specificity of AGE-RAGE imbalance in IPF [42, 43]. Therefore, our study aims to evaluate the potential utility of serological AGEs and sRAGE levels in IPF.

## Methods

### Ethical statement and patient recruitment

Patients were recruited in the outpatient Unit of ILDs from University Hospital of Bellvitge: 62 IPF, 22 cHP, and 20 fNSIP. The patient’s diagnosis was discussed in the multidisciplinary committee and was established in accordance with the American Thoracic Society/European Respiratory Society criteria [5, 12, 44]. Twelve healthy subjects age-matched were recruited as control. The inclusion criteria of healthy volunteers were the absence of chronic illness and pulmonary functional test abnormalities. We collected serum samples after 3 years from 23 IPF patients in order to analyze the changes of AGEs-sRAGE levels and the progression of the disease. Lung transplantation and mortality were reported. These patients and those that return to their original care centers were excluded for the second serological evaluation.

This observational prospective study was approved by the Ethics Committee of University Hospital of Bellvitge (CEIC, ref. PR082/15) and all patients signed the written informed consent before their inclusion.

### Pulmonary function test (PFT)

Spirometry, lung volume, and Diffusing Capacity of Lung for Carbon Monoxide (DLCO) were measured in the Medisoft® bodybox Plethysmograph at the PFT laboratory. Three reproducible spirometry measurements were performed (with a difference of less than 150 ml between each one) to obtain the Forced Vital Capacity (FVC) and Total Lung Capacity (TLC). Two maneuvers were recorded to register the best value by DLCO single-breath technique.

### Sample collection, processing and measurement

Peripheral blood samples were collected from participants in a BD Vacutainer® SST™ tubes (Becton, Dickinson and Company, USA), and serum faction were obtained using standardized procedures. AGE and sRAGE were measured by specific commercial ELISA kit; Human RAGE Quantikine ELISA Kit (DRG00; R&D Systems, USA) and Human AGEs ELISA Kit (CSB-E09412h; Cusabio Biotech Co., China), following the manufacturer’s recommendations.

### Statistical analysis

The results were expressed as mean [SD] or median [interquartile range] and were compared using one-way ANOVA or Student’s t test, followed by the appropriate post hoc analysis.

To evaluate the power of AGEs/sRAGE to discriminate between the fibrotic ILDs, receiver operating characteristic (ROC) analyses were performed: the levels of AGE and sRAGE in blood serum were considered as a continuous variable and the diagnostic classification was accepted as a dichotomous variable. Cutoff points were calculated by the ROC curves with the highest possible sensitivity and specificity, and their discriminative potential was quantified following 3 different diagnostic accuracy measures: Diagnostic effectiveness (Accuracy), Likelihood ratio for positive test results (LR+), and Youden’s index (J). To determine the serum levels that predict 3-year survival rates in the whole IPF cohort, ROC curve analysis was also performed, and the rate was estimated by Kaplan–Meier analysis using log-rank test as statistic contrast. In addition, Pearson’s correlations were performed to test the relation among PFT, sRAGE and AGE serum levels at the beginning of the study and at the end of the 3-year follow-up. Statistical software SPSS statistic 24 (IBM, USA) was used for statistical analyses. Significant differences were accepted when the *p* value was < 0.05 (*) or < 0.01 (**).

## Results

### Clinical characteristics

Clinical features of IPF, cHP, fNSIP and control groups are included in Table 1. IPF patients were predominantly males and former smokers. No statistical differences among the studied groups were found in age, pack-years, nor smoking cessation. Regarding PFTs, control subjects showed better results in the percentage of predicted FVC, DLCO and TLC than fibrotic ILDs, as expected (*p* < 0.01).

**Table 1:** Group features

Patient characteristics	Control	IPF	cHP	fNSIP
Subjects (n)	12	62	22	20
Gender (Male/Female)	9 / 3	53 / 9	10 / 12	5 / 15
Age (yr.)	69.11 ± 5.97	68.43 ± 8.57	64.74 ± 11.36	63.33 ± 11.62
Smoking (Former/Never)	5 / 7	43 / 19	12 / 10	5 / 15
Pack-years	20.23 ± 26.07	31.28 ± 20.74	19.25 ± 27.39	20.70 ± 11.67
Smoking cessation (yr.)	7.67 ± 9.07	15.17 ± 8.69	18.56 ± 10.38	9.00 ± 4.58
% FVC	116.20 ± 14.23**	78.71 ± 20.33	82.59 ± 15.98	81.40 ± 25.93
% DLCO	103.10 ± 2.62**	50.41 ± 20.75	56.98 ± 19.40	59.11 ± 27.67
% TLC	112.75 ± 18.31**	79.79 ± 18.69	80.88 ± 17.82	83.61 ± 19.90

Data represent the mean ± SD, *cHP* chronic hypersensitivity pneumonitis, *DLCO* diffusing capacity for carbon monoxide, *fNSIP* fibrotic non-specific interstitial pneumonia, *FVC* forced vital capacity, *IPF* idiopathic pulmonary fibrosis, *TLC* Total lung capacity. (**) *p*-value < 0.01

### AGEs and sRAGEs serum concentration in pulmonary fibrosis

**Fig. 1:**
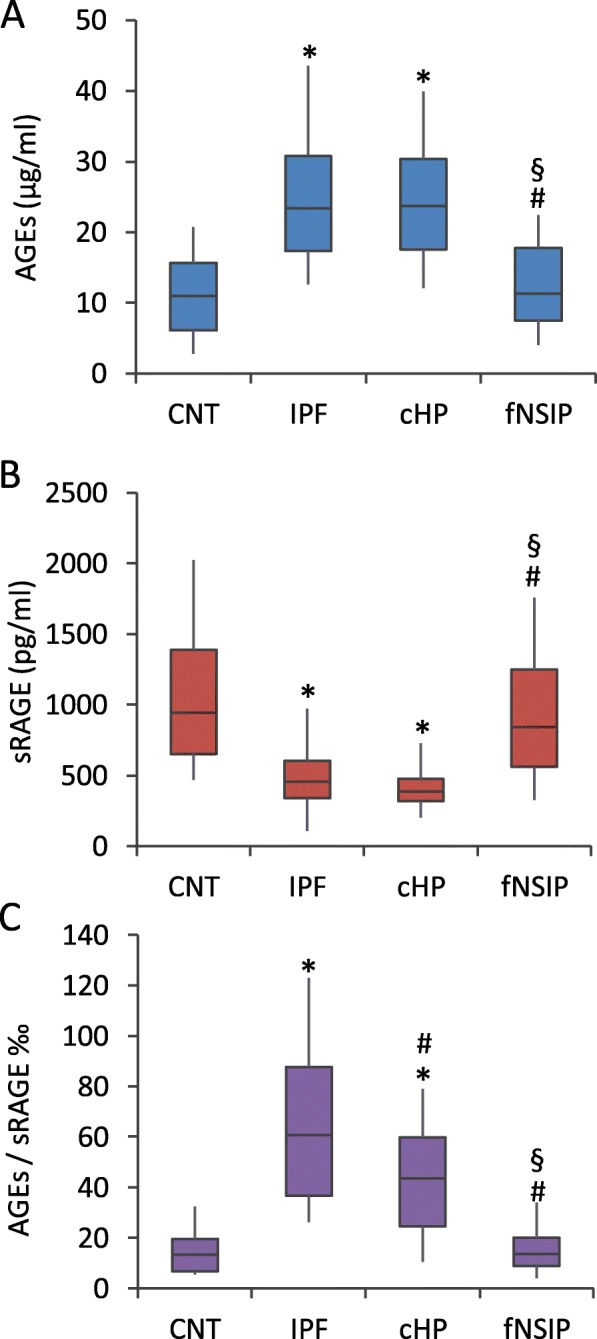
ELISA of AGEs and sRAGE in serum blood from controls (CNT) and fibrosing ILDs. **a** and **b** ELISA for total AGEs and sRAGE showed significant differences between control group with IPF and cHP (*p < 0.01), and differences between fNSIP patients with IPF (#*p* < 0.01) and cHP (§*p* < 0.01) groups. However, there were no significant differences between IPF with cHP and control with fNSIP. **c** AGEs/sRAGE ratio estimated by ELISA was greater in IPF and cHP groups compared with control (*p < 0.01). The ratio also allowed to distinguish between IPF patients with cHP and fNSIP (#*p* < 0.05), and cHP with fNSIP group (§*p* < 0.01), whereas this did not distinguish between control and fNSIP patients. Data were analyzed by one-way ANOVA following by the appropriate post-hoc analysis

AGEs levels were significantly higher in serum samples from IPF and cHP compared with control and fNSIP patients (*p* < 0.01). There were no statistical differences in AGEs serum levels between IPF and cHP groups, and no differences were observed when fNSIP was compared to control (Fig. 1a).

On the other hand, a significant decrease in sRAGE concentration was observed in serum samples from IPF and cHP patients compared to fNSIP and control donors (*p* < 0.01). There were no significant differences comparing IPF and cHP (Fig. 1b).

The serum AGE/sRAGE ratio of each patient was analyzed from the ELISA results. An increase of 4.20 and 4.48-fold was observed in IPF patients compared with fNSIP and control subjects, respectively (*p* < 0.01). Furthermore, the ratio was statistically significant higher in IPF patients than cHP patients (*p* < 0.05). In addition, the AGE/sRAGE ratio was 2.84-fold higher in cHP as compared to fNSIP (*p* < 0.01). AGE/sRAGE ratio in fNSIP was similar to the control group (Fig. 1c).

### AGE and sRAGE as a discriminative factor between IPF and fNSIP

**Fig. 2:**
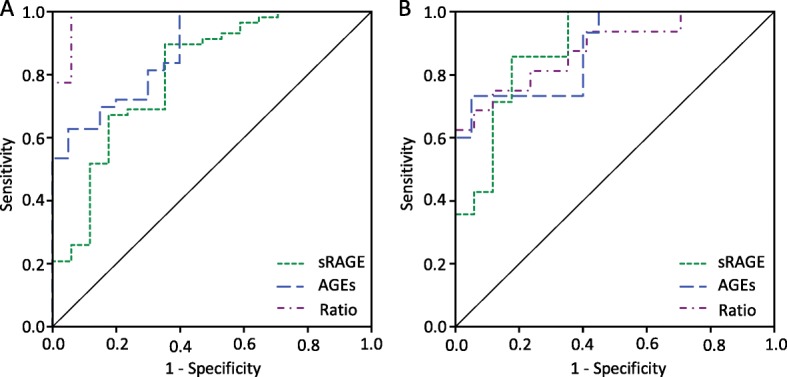
ROC curve for total AGEs, sRAGE levels and Ages/sRAGE ratio. **a** AGEs and sRAGE showed a good capability to differentiate between IPF patients and fNSIP group (AUC = 0.879 and 0.801, respectively), whereas the AGEs/sRAGE ratio had higher predictable capability (AUC 0.987, CI = 0.959–1.000) to discriminate between both. **b** Comparing cHP and fNSIP, all the three indicators (AGEs, sRAGE levels and AGE/sRAGE ratio) showed AUC values quite good to be considered diagnostic tests (AGEs = 0.883, sRAGE = 0.887, Ratio = 0.882). Data were evaluated by ROC analyses; blood serum levels of AGE and sRAGE were considered as a continuous variable, and the diagnostic classification was accepted as a dichotomous variable

We performed a ROC curve analysis to evaluate the potential IPF specificity of AGEs and sRAGE levels in blood serum (Additional file 1). The results showed that serum sRAGE and AGEs levels present a high capacity to differentiate IPF patients from fNSIP (AUC = 0.801 and 0.879, respectively), and between cHP and fNSIP patients (AUC = 0.887 and 0.883, respectively) (Fig. 2a, b). Furthermore, the AGE/sRAGE ratio also distinguished between fNSIP and IPF (AUC = 0.987, CI = 0.959–1.000), and between cHP and fNSIP (AUC = 0.882, CI = 0.766–0.998).

On the other hand, AGEs and sRAGE levels did not demonstrate capability to differentiate IPF and cHP (AUC = 0.383 and 0.507, respectively), while ratio showed very low differential power (AUC = 0.713, CI = 0.569–0.856).

To evaluate the discriminative potential of AGEs, sRAGE and AGE/sRAGE, optimal cut-off points were obtained from the ROC curve. Table 2 showed that AGEs/sRAGE ratio had a higher diagnostic effectiveness (accuracy = 98.28%, LR+ = 17.00, J = 0.94) than serum levels of AGEs and sRAGE separate values to distinguish between IPF and fNSIP patients. Moreover, although the diagnostic accuracy and the Youden’s index of the three parameters were quite similar, serum levels of AGEs were more appropriate than sRAGE and the AGEs/sRAGE ratio to differentiate between cHP and fNSIP (LR+ = 14.67, 4.86, and 6.38; respectively).

**Table 2:** Cut-off values and diagnostic accuracy measures of AGEs, sRAGE and AGEs/sRAGE ratio

	IPF – fNSIP			cHP – fNSIP		
Indicators	AGE	sRAGE	Ratio	AGE	sRAGE	Ratio
Cut-off value	19.25 μg/ml	782.59 pg/ml	25.65 ‰	20.92 μg/ml	529.57 pg/ml	24.87 ‰
Sensitivity (%)	69.77	89.66	100.00	73.33	85.71	75.00
Specificity (%)	85.00	64.71	94.12	95.00	82.35	88.24
Accuracy (%)	74.60	84.00	98.28	85.71	83.87	81.82
LR+	4.65	2.54	17.00	14.67	4.86	6.38
J	0.55	0.54	0.94	0.68	0.68	0.63

*AGE* advanced glycation end-product, *cHP* chronic hypersensitivity pneumonitis, *fNSIP* fibrotic non-specific interstitial pneumonia, *J* Youden’s index, *LR+* Likelihood ratio for positive test results, *IPF* idiopathic pulmonary fibrosis, *sRAGE* soluble receptor for advanced glycation end-products

### AGE and sRAGE correlation with PFTs

**Fig. 3:**
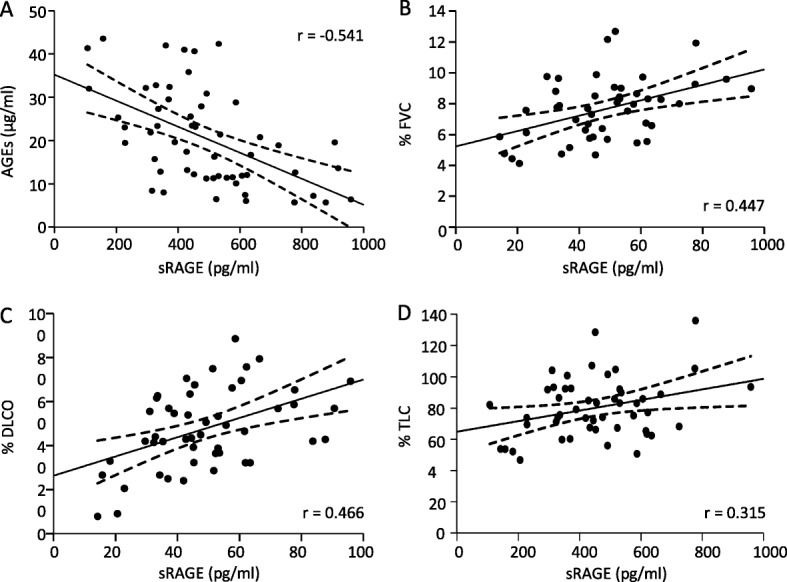
Pearson’s correlation between sRAGE, AGEs and PFT in IPF patients. **a** Results showed a negative relationship between sRAGE and AGEs in serum samples from IPF patients. **b, c, d** There was a positive correlation between sRAGE serum levels and percentages values of FVC, DLCO and TLC in IPF samples. The lineal relationship between the variables was analyzed by Pearson’s correlations coefficient

Pearson’s test showed that increased levels of AGEs correlated with lower sRAGE in IPF patients (*r* = − 0.541, *p* < 0.01) (Fig. 3a). Meanwhile, cHP patients also showed a negative correlation (*r* = − 0.496, *p* < 0.05), fNSIP patients did not show any significant correlation (Additional file 2).

Pearson’s test also showed that lower levels of sRAGE in serum samples from IPF patients correlated with worse lung functionalism: % predicted of FVC (*r* = 0.447,* p* < 0.01), DLCO (*r* = 0.466, *p* < 0.01) and TLC (*r* = 0.315; *p* < 0.05) (Fig. 3b, c, d). However, the concentration of AGEs did not show any correlation with PFTs in IPF (data not shown). Regarding cHP and fNSIP samples, there was also a positive correlation between sRAGE and PFTs, whereas AGEs levels did not show any correlation in either entity (cHP and fNSIP).

### AGEs-sRAGE concentration in the follow-up of IPF patients

**Fig. 4:**
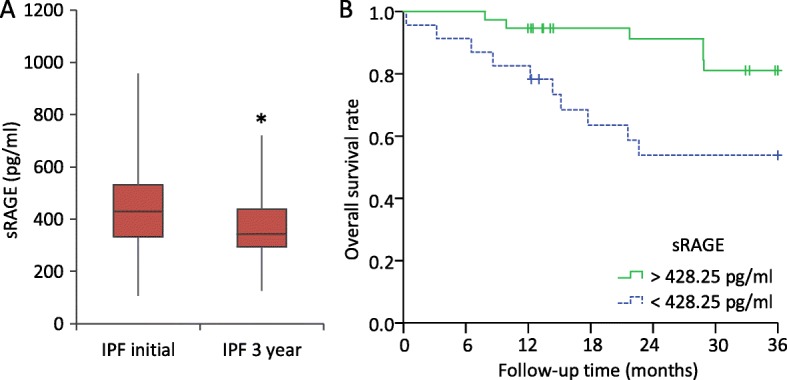
Serum levels and predictive capability of sRAGE for 3-year survival rates. **a** Results showed a significant decrease of sRAGE in IPF patients beyond 3-years. **b** Kaplan–Meier analysis showed IPF patients with sRAGE levels in serum under 428.25 pg/ml had lower survival rate (*p* = 0.014) at 3-yr follow-up. Comparison of AGEs/sRAGE levels in follow-up were analyzed by Paired sample t-test, the threshold of sRAGE levels in blood were calculated by ROC analysis and survival rate was estimated by Kaplan–Meier analysis using log-rank test as statistic contrast

Twenty-three IPF patients were re-evaluated 3-years after their inclusion. Table 3 show a decrease in the lung functional values, with a significant decrease in the mean FVC. Regarding ELISA analyses, only sRAGE serum levels showed a significant decrease between the basal level and 3-years after (*p* < 0.05) (Fig. 4a).

**Table 3:** ELISA measurements and lung function from IPF patients at 3-year interval

Patient characteristics	Initial	3 yrs.	*p*-value
sRAGE (pg/ml)	461.53 ± 191.77	375.78 ± 143.25	0.029*
AGEs (μg/ml)	19.07 ± 8.63	17.19 ± 8.45	0.340
Ratio (‰)	52.58 ± 34.58	41.13 ± 14.91	0.079
% FVC	89.21 ± 19.50	79.14 ± 20.63	0.001**
% DLCO	57.84 ± 11.54	48.68 ± 12.73	0.000**
% TLC	87.22 ± 16.89	76.47 ± 15.57	0.000**

Data represent the mean ± SD, *AGE* advanced glycation end-product, *DLCO* diffusing capacity for carbon monoxide, *FVC* forced vital capacity, *sRAGE* soluble receptor for advanced glycation end-products, *TLC* Total lung capacity. (*) *p*-value < 0.05, (**) *p*-value < 0.01

In addition, the ROC analysis of the whole IPF group showed sRAGE levels as a reliable predictor of the 3-year follow-up (AUC = 0.724, 95% CI = 0.586–0.863, *p* < 0.01). Survival estimation by Kaplan–Meier was lower in IPF patients with serum concentrations of sRAGE under 428.25 pg/mL at the basal level (*p* = 0.014) (Fig. 4b).

**Fig. 5:**
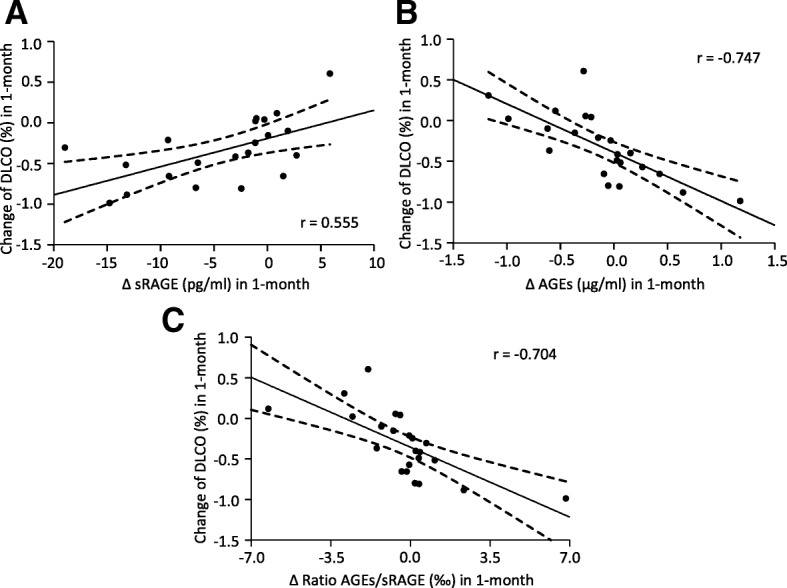
Correlation between changes in DLCO and differences in sRAGE-AGEs levels in IPF patients. **a** In IPF patients, a decline of DLCO was related with a decrease of sRAGE in serum (*r* = 0.555, *p* < 0.01). **b and c** On the contrary, AGEs levels and the AGEs/sRAGE ratio were negatively correlated with the percentage of changes in DLCO in IPF patients (*r* = − 0.747 and − 0704, respectively, *p* < 0.01), meaning poor lung function in patients with an increase of AGEs-sRAGE imbalance over time. The changes in serum sRAGE and AGEs and lung function from IPF patients was calculated, adjusting over time: Δ ELISA in a month = [ELISA final – ELISA initial]/month; change of % predicted PFT = [PFT final – PFTs initial]/month. Pearson’s correlation coefficients were performed to analyze the lineal relationship between the variables

Furthermore, we correlated the changes in serum sRAGE and AGEs from IPF patients adjusted over time (Δ ELISA in a month = [ELISA final – ELISA initial]/month) with the percentage of change of lung function in the same interval (change of % predicted PFT = [PFT final – PFTs initial]/month). The results show a relationship between variations in sRAGE, AGEs and their ratio levels with the lung functional values (% predicted) of FVC, TLC and DLCO (Table 4). IPF patients with a decrease in sRAGE serum levels during the follow-up usually showed a significant decline in % predicted DLCO as well (Fig. 5a). On the other hand, an increase of AGEs serum levels was highly associated with a DLCO decline, while no changes in DLCO associated no changes in AGEs serum levels (Fig. 5b).

**Table 4:** Correlation between changes in ELISA measurements and lung function

Correlations	Change of PFT	r	R^2^	*P*-value
Δ sRAGE	FVC	0.437	0.191	0.042*
	DLCO	0.586	0.343	0.005**
	TLC	0.412	0.170	0.057
Δ AGEs	FVC	−0.452	0.204	0.027*
	DLCO	−0.747	0.557	0.000**
	TLC	−0.486	0.237	0.022*
Δ Ratio	FVC	−0.479	0,230	0.018*
	DLCO	−0.704	0.495	0.000**
	TLC	−0.352	0.124	0.100

*AGE* advanced glycation end-product, *DLCO* diffusing capacity for carbon monoxide, *FVC* forced vital capacity,* PFT* pulmonary function test, *r* correlation coefficient, *R*^*2*^ coefficient of determination, *sRAGE* soluble receptor for advanced glycation end-products, *TLC* Total lung capacity. (*) *p*-value < 0.05, (**) *p*-value < 0.01

With regards to the AGEs/sRAGE ratio, a negative correlation with % predicted DLCO was observed as well. Therefore, IPF patients with an increase of the AGEs/sRAGE ratio had a more rapid decline in DLCO (Fig. 5c). Furthermore, an increment of AGEs in serum was correlated with a loss of their receptor in IPF patients, maintaining a negative correlation over time (Additional file 3).

## Discussion

Some reports have shown an association between the blood sRAGE levels and the state of alveolar epithelium and lung injury [40, 45, 46]. Furthermore, previous results from our group found AGEs increased in IPF lungs [20]. The present results suggest that serum AGEs and sRAGE are different in IPF than in fNSIP, and their ratio is even higher than in cHP, which could be a useful information for the differential diagnosis of these fibrotic ILDs. Moreover, low levels of sRAGE at diagnosis predict poor survival in IPF. Finally, the AGE and RAGE changes correlate with lung functional changes over time.

While the radiological fNSIP pattern may be similar to IPF, the histological pattern is completely different. Therefore, lung biopsy is required in some cases to differentiate both entities. In this case, finding biological lung markers that could be measured in blood samples to differentiate both entities would be of interest for the clinical practice. Serum AGEs and sRAGE show a completely different pattern in IPF and fNSIP, probably due to the lack of fibroblastic foci and honeycombing in fNSIP lungs and the pathogenic differences [11]. Lung RAGEs are expressed in alveolar epithelial cells and lungs with usual interstitial pneumonia pattern show lower RAGEs expression than NSIP lungs [20]. Manichaiku et al. showed lower plasma levels in IPF and HP patients than control subjects [47]. The decrease of RAGEs is not present in all fibrotic ILDs; fNSIP presents serum RAGEs and AGEs similar to normal subjects.

Lungs are the main source of RAGEs in healthy conditions [36]. Several studies concluded that cleaving full length RAGE (FL-RAGE), the transmembrane form, is the main way to produce the soluble form of alveolar epithelial cells (AECs) from healthy human lungs [48]. This might suggest that decrease in sRAGE found in IPF patients could be interpreted as a lack of RAGE synthesis or the loss of AECs. Thus, variations in the serum sRAGE levels would reflect the possible changes in lung function. The association between serum sRAGE and PFTs, as well as the changes over time would support this hypothesis. Longitudinal changes in FVC and DLCO have been found to have important prognostic value in IPF [49]. However, predicting prognosis at diagnosis remains a challenge. In our IPF cohort, patients with lower levels of sRAGE at the beginning of this study (under 428.25 pg/mL) showed worse lung-transplant progression free survival rate at 3 years, in accordance with previous observations from Yamaguchi and colleagues [37].

The decrease of sRAGE might be related to the progressive loss of alveolar structures in lung fibrosis lung. Chronic lung diseases that cause a progressive destruction of the alveolar structures such as chronic obstructive pulmonary disease (COPD) showed low levels of sRAGE compared with controls [39, 50]. Those results have been correlated with a deterioration in lung function over time. Similarly, mechanical damage of lung showed low levels of sRAGE [41, 51]. On the other hand, the downregulation of RAGE has also been related to several pro-fibrotic pathways [52, 53]. There are some reports that showed a predisposition to develop a spontaneous pulmonary fibrosis in RAGE null mice [54]. All these findings suggest that the destruction of alveolar structures by fibrotic changes might favor the loss of RAGEs in IPF patients, and this downregulation of sRAGE, at the same time, could favor the fibrosis progression.

Regarding AGEs levels in serum, although a significant increase has been found in IPF and cHP compared to fNSIP, the measurement in the IPF group at the beginning of the study was not associated with pulmonary functional values. However, changes in AGEs levels over time were associated with FVC, TLC and DLCO decline. AGEs are increased in IPF lungs and have been associated with myofibroblast formation and ECM stiffness in vitro [55]. In addition, if sRAGE decreases, the pro-fibrotic effect of AGEs could increase due to the decreased decoy effect [56]. Our results also showed that those IPF patients with higher AGEs/sRAGE ratio showed a faster decline in DLCO. These findings indicate that an increase of AGEs or the AGEs/sRAGE ratio might indicate the degree of remodeling changes due to pulmonary fibrosis.

Some limitations of the study are the small number of the included cases in each group and the lack of validating cohort. Furthermore, no other possible biomarkers have been measured in our cohort in order to compare the differences or supplementary value for diagnostic and prognostic purposes. However, the results are useful for inclusion of this biomarker in longitudinal multicenter studies to evaluate potential biomarkers in fibrotic ILDs.

In the last few years, extensive reviews had been done to evaluate the multiple potential serum markers proposed, some of them implied in ECM remodeling or AEC dysfunction, suggesting a much better diagnostic accuracy and practicality when they were evaluated together [57–59]. Our model base on AGEs-sRAGE estimation and ratio also emphasizes the need to mix biomarkers for improving predictive power.

## Conclusions

In conclusion, these results demonstrate that the ratio AGEs/sRAGE could be considered as a new potential serum biomarker for aiding in the differential diagnosis between the most common fibrosing ILDs, although a prospective multicenter study for validating this pivotal results would be required. Furthermore, serum sRAGE level is a prognostic biomarker in IPF.

## Additional files

Additional file 1: ROC statistics for sRAGE and AGE serum levels. AGE: advanced glycation end-product, AUC: area under the curve, cHP: chronic hypersensitivity pneumonitis, fNSIP: fibrotic non-specific interstitial pneumonia, CI: confidence interval, IPF: idiopathic pulmonary fibrosis, sRAGE: soluble receptor for advanced glycation end-products, ROC: Receiver operating characteristic. (*) *p*-value < 0.05, (**) *p*-value < 0.01. (XLSX 9 kb)

Additional file 2: Correlation between RAGE, AGE and PFT. AGE: advanced glycation end-product, cHP: chronic hypersensitivity pneumonitis, DLCO: diffusing capacity for carbon monoxide, fNSIP: fibrotic non-specific interstitial pneumonia, FVC: forced vital capacity, IPF: idiopathic pulmonary fibrosis, r: correlation coefficient, R^2^: coefficient of determination, sRAGE: soluble receptor for advanced glycation end-products, TLC: Total lung capacity. (*) *p*-value < 0.05, (**) *p*-value < 0.01. (XLSX 9 kb)

Additional file 3: AGEs-sRAGE correlation in IPF patients during follow-up. IPF patients with a decline of AGEs in serum showed an increase of soluble fragment of the receptor in blood. (PDF 109 kb)

## Abbreviations

AECs: Alveolar Epithelial Cells
AGEs: Advanced glycation end-products
AUC: Area Under the Curve
cHP: Chronic Hypersensitivity Pneumonitis
COPD: chronic obstructive pulmonary disease
DLCO: Diffusing Capacity for Carbon Monoxide
ECM: Extracellular Matrix
FL-RAGE: Full-length RAGE
fNSIP: Fibrotic non-specific interstitial pneumonia
FVC: Forced Vital Capacity
ILDs: Interstitial Lung Diseases
IPF: Idiopathic Pulmonary Fibrosis
J: Youden’s index
LR+: Likelihood ratio for positive test results
PFT: Pulmonary Function Test
RAGE: Receptor for AGEs
ROC: Receiver Operating Characteristic
sRAGE: Soluble RAGE
TLC: Total Lung Capacity
UIP: Usual Interstitial Pneumonia
