# Supplementary figures and images for: Serum AGE/RAGEs as potential biomarker in idiopathic pulmonary fibrosis
Source: Respir Res. 2018 Nov 8;19:215. doi: 10.1186/s12931-018-0924-7 (PMC6225674; doi:10.1186/s12931-018-0924-7)

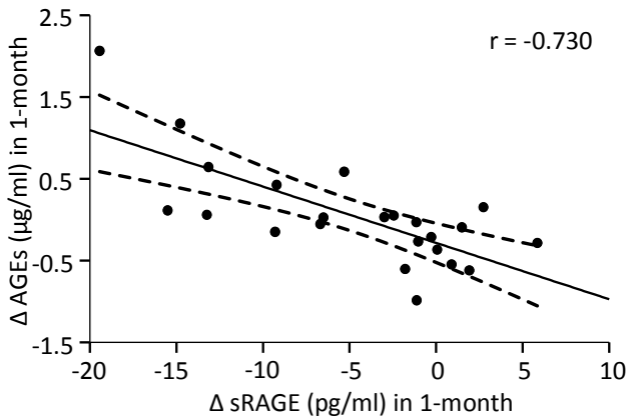

Supplement: Supplementary file 3 — AGEs-sRAGE correlation in IPF patients during follow-up. IPF patients with a decline of AGEs in serum showed an increase of soluble fragment of the receptor in blood. (PDF 109 kb) [file 12931_2018_924_MOESM3_ESM.pdf]
